# A free-geometry geodynamic modelling of surface gravity changes using Growth-dg software

**DOI:** 10.1038/s41598-021-02769-z

**Published:** 2021-12-06

**Authors:** Antonio G. Camacho, Peter Vajda, Craig A. Miller, José Fernández

**Affiliations:** 1 Instituto de Geociencias (CSIC, UCM), C/Doctor Severo Ochoa, 7, 28040 Madrid, Spain; 2 Earth Science Institute, Slovak Academy of Sciences, Dubravska cesta 9, P.O. Box 106, 840 05 Bratislava, Slovakia; 3 GNS Science, Wairakei Research Center, Private Bag 2000, Taupō, 3330 New Zealand

**Keywords:** Environmental sciences, Natural hazards, Solid Earth sciences, Mathematics and computing

## Abstract

Globally there is abundant terrestrial surface gravity data used to study the time variation of gravity related to subsurface mass and density changes in different geological, geodynamical and geotechnical environments. We present here a tool for analysing existing and newly acquired, 4D gravity data, which creates new findings from its reuse. Our method calculates in an almost automatic way the possible sources of density change responsible for the observed gravity variations. The specifics of the new methodology are: use of a low number of observation points, relatively small source structures, low signal/noise ratio in the data, and a free 3D source geometry without initial hypothesis. The process is based on the non-linear adjustment of structures defined by aggregation of small cells corresponding to a 3D section of the sub-floor volume. This methodology is implemented in a software tool, named GROWTH-dg, which can be freely downloaded for immediate use, together with a user manual and application examples.

## Introduction

The precision of relative gravity measurements, on the order of a few μGal (for instance, about 6 μGal for the Scintrex and about 11 μGal for LCR meter, in ideal field conditions) offers application for geodynamic purposes^1^. Measurement and interpretation of spatio-temporal gravity changes has been used in several geodynamics contexts: seismology^2,3^, gas extraction^4^, hydrology^5–7^, CO_2_ sequestration^8^ and Earth’s dynamics^9^. However, the geodynamic context with a large number of publications is volcanology^10–22^. These gravimetric measurements, often combined with collocated displacement measurements, allow for a detailed interpretation of the source processes.

Most of the processes to recover the structures with temporal density changes responsible for the observed gravity changes, are based on prescribed analytic solutions for source bodies of simple geometry such as spheres, ellipsoids, disks or prisms representing sills or dykes^12,23^. For many cases these solutions are approximate but informative, given the usually low number of observations (10–20) and low model degrees of freedom (typically < 10 parameters). However, for those cases with larger number of observations, a more ambitious approach can be applied: a 3D modelling without a priori assumption about geometrical patterns. Those free-geometry inversion methods are more developed for structural studies upon large number of gravity anomaly data, the complete Bouguer anomaly (CBA), or data from gravity gradient components^24^. A way of classifying gravity inversion methods is to consider the unknown type. Some studies (linear approach) consider density values as the unknown model parameters^25^. These methods require additional constraints to get sharp discontinuity limits for anomalous bodies^26^. For other approaches, the parameters to be adjusted are geometric (non-linear approach): depth of a discontinuity surface^27–29^, polyhedral structures^30,31^, aggregation of small cells^32^. Binary or multinary approaches^33^ could be considered as intermediate.

Camacho et al.^34^ updated a methodology, software (named Growth) to carry out a nearly automatic 3D inversion of CBA gravity data in the context of structural studies. It can provide, without specific assumptions, anomalous density models suitable for different environments, including effects for general downward density increase. Here we present a modification of this methodology specifically for inverse modelling the 4D microgravity changes, in particular the sparse and inaccurate microgravity data given on the topographic surface. The present approach produces similar results to multinary approach but on a rather easy way.

The main differences between inverting the CBA gravity data and the 4D microgravity are: (a) the number of data points (e.g., some tens for microgravity vs several hundred or more for CBA data); (b) the signal/noise ratio (e.g., as much as 80% for CBA vs 30% for gravity changes); and (c) the density source structure (e.g., isolate, rather simple and small for changes vs. very extended, stratified and complex for CBA).

We also present the corresponding new and freely available (open source) software in which this methodology is implemented, including its testing using some synthetic cases and an application test case as a real world example. Both, the methodology and software, together with the used data, are described in the Methods section.

The essential differences between Growth and Growth-dg are:

– Methodological differences: (1) Inclusion of heterogeneous models, (2) new criterion for process end, and (3) some small adaptations of the approach to work with few data points and low correlated signal.

– Code differences: (1) It is much more simple for execution, and (2) it offers a lot of new figures which help for decisions about the modelling parameters.

## Results

### Synthetic cases

Here we illustrate aspects of the inversion method using several synthetic examples. We refer these synthetic cases to a site with mild relief and an average diameter of 12 km (a similar size to that in the final real test case). We also include synthetic examples that are not particularly favourable to the inversion approach, to provide a realistic perspective on the modelling.

**Figure 1:**
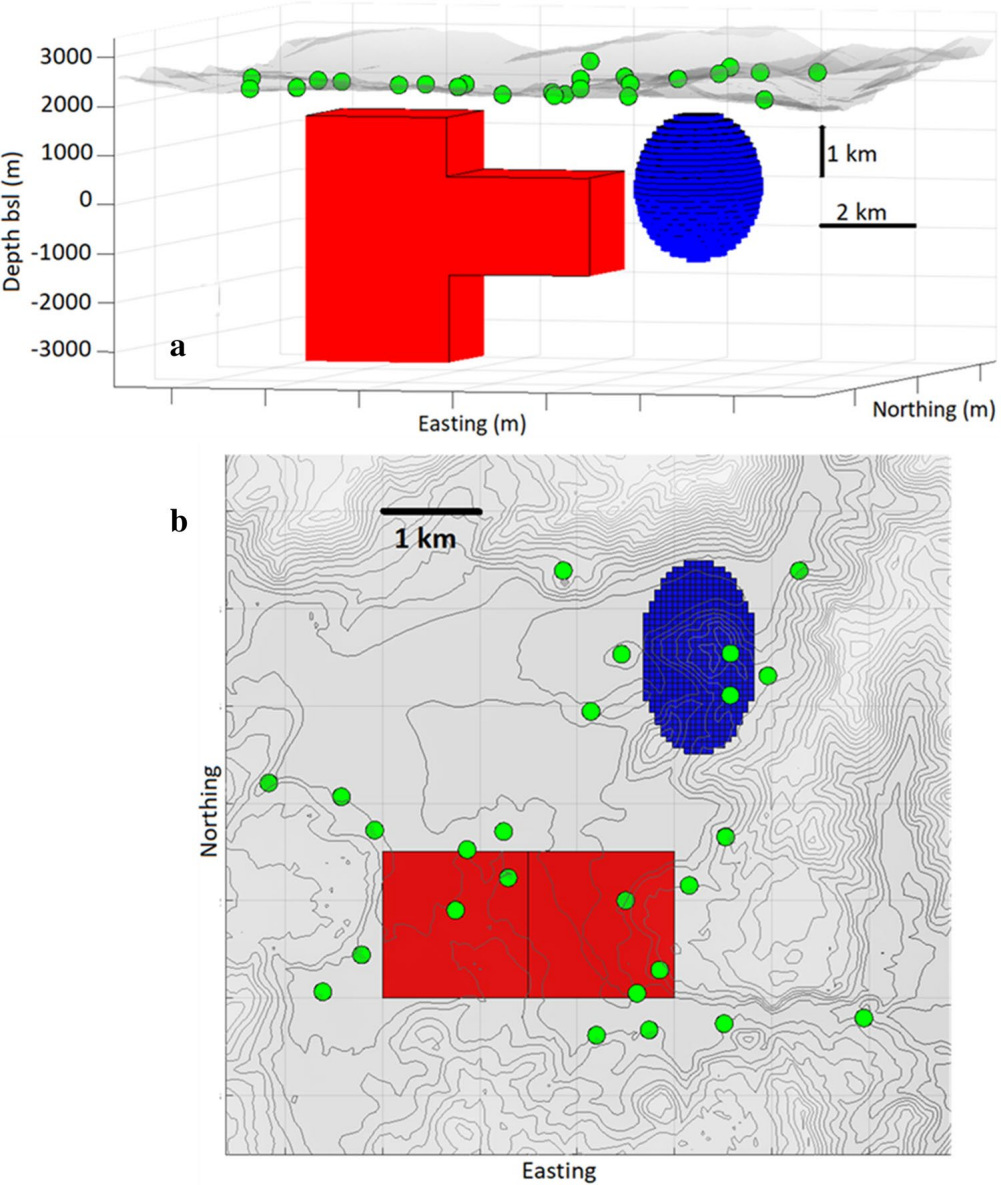
Synthetic model of positive and negative anomalous density bodies buried below the test area. The red T-shape body has a positive density contrast (+ 10 kg/m^3^). The blue ellipsoidal body has a negative density contrast (−15 kg/m^3^). (**a**) Perspective 3D view, (**b**) Plan view. Green circles denote simulated gravity stations. The topography is shown in grey color on both panels. Matlab (https://www.mathworks.com) software was used to create this figure.

We consider a synthetic source structure (Fig. 1) composed of two anomalous bodies: (1) a positive density contrast (+ 10 kg/m^3^) body composed of two parallelepiped prisms (a T-shape body) with depth ranging from 500 to 5500 m below the surface topography level (from + 2500 m to −3500 m in altitude), and (2) a negative density contrast (−15 kg/m^3^) triaxial ellipsoid (semi-axes of 1000, 1500 and 2000 m) and mean depth 2000 m below surface topography level. These synthetic bodies are created to represent fault zoned (parallelepiped) or magma reservoirs (ellipsoid), features commonly found in volcanic regions. They could also represent other similar shaped geologic structures including dykes, or geothermal reservoirs. The density changes are typical of those caused by migrating aqueous liquids and can represent temporal density changes between the observation intervals.

**Figure 2:**
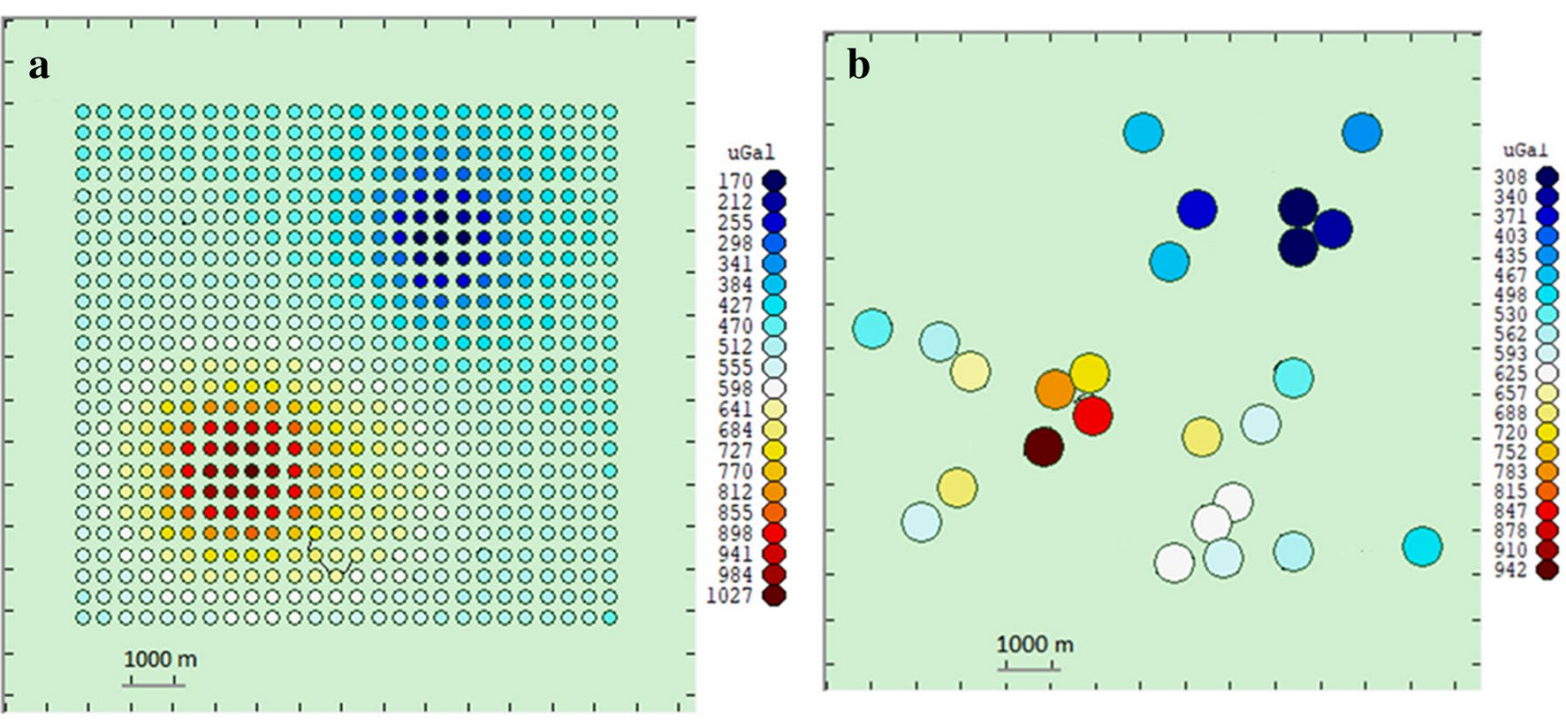
Calculated gravity values corresponding to the synthetic structure of Fig. 1 plus an additional offset of 500 μGal. (**a**) On a grid of 660 observation points. (**b**) At 24 simulated gravity stations. This figure was created using Growth-dg software.

To create a synthetic dataset, we compute theoretically the gravity response of these two bodies (by decomposing them into small prims and applying Eq. (1) in “Methods” section for direct computation), on a grid of 660 locations and at 24 scattered locations. (Fig. 2). To test an aspect of our methodology for handling offsets in a dataset we also include an example where an offset value of 500 μGal is introduced to the data.

Next, we show the results of the application of our new inversion approach to the synthetic data set. The software partitions the subsurface volume into 90,000 small parallelepiped cells with an average side of about 200 m. As shown in the Method section, there are two parameters whose values determine the adjusted model. They are the relative model volume *R* (as % of the available subsurface volume) and the balance fitting-smoothing value *λ,* required by the regularization conditions. We simulate different scenarios, to test the ability of the inversion method in different situations. The studied scenarios are:

- i. Large exact data set + only negative (rather rounded) source body.
- ii. Large exact data set + only positive (rather angulate) source body.
- iii. Large exact data set + combined positive and negative sources.
- iv. Large inexact data set + combined source bodies.
- v. Small exact data set + combined source bodies.
- vi. Small inexact data set + combined source bodies.

The “large data set” denotes large number of gravity data on a regular grid with a relatively small inter-station spacing (Fig. 2a). The “small data set” denotes gravity data given at relatively small number (24) of gravity stations irregularly scattered over the relief (Fig. 2b).

For the large data set scenarios, the inversion recovers the model geometry more accurately, than for the small data set scenarios. This is not caused by defects in the inversion process, but rather by the loss of relevant information between large datasets with low error and small datasets with high error.

Cases i, ii, iii and iv have a large set (660 points) of closely spaced (120 m) observations on a regular grid (Fig. 2a). This is a much more favourable situation than that corresponding to real cases for which the number of gravity stations is usually a few dozens. However, it helps to illustrate the reliability of inversion process under good conditions.

**Figure 3:**
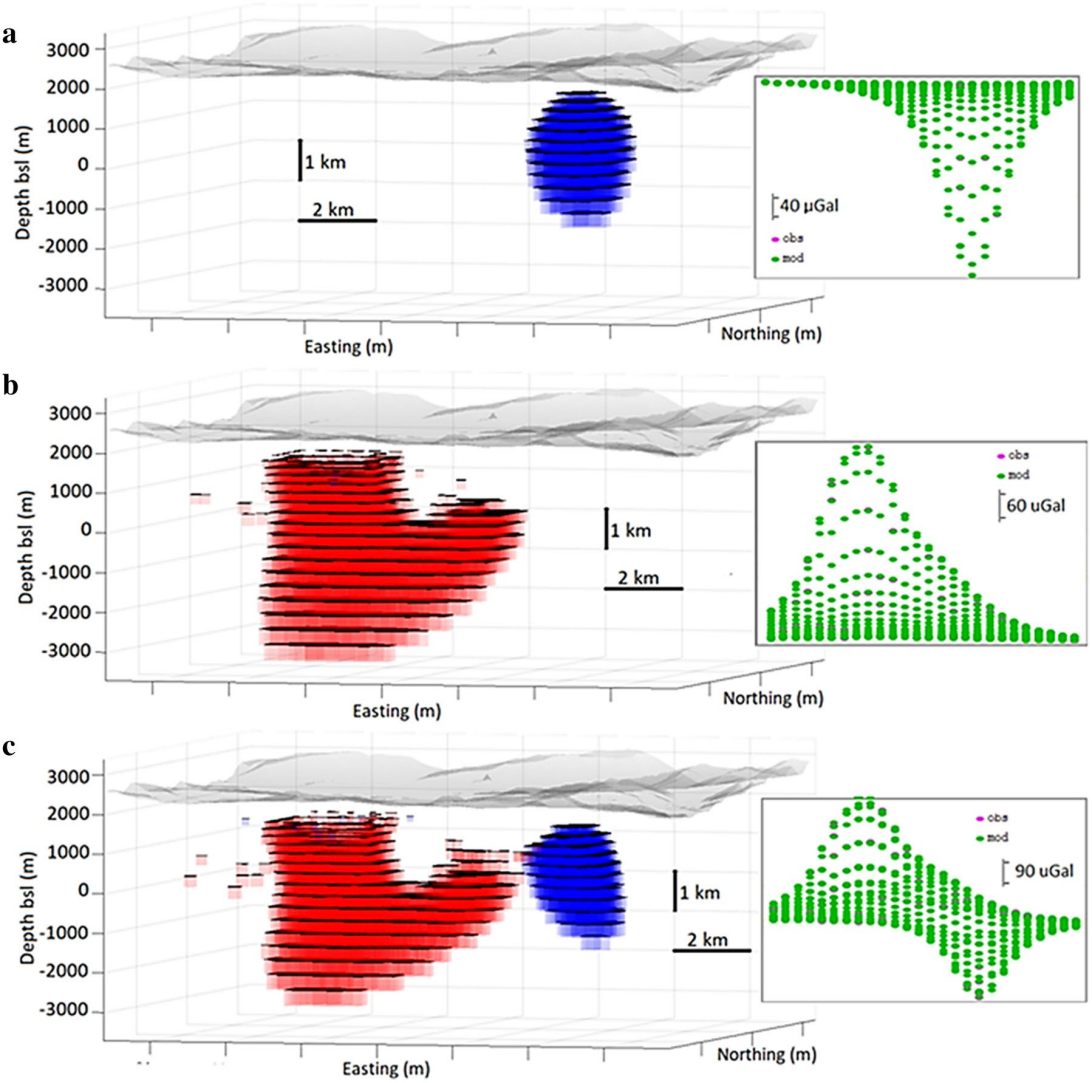
Inversion models for synthetic exact data over a large set of data points (Scenarios i, ii, iii). (**a**) Model of negative density anomaly body. (**b**) Model of positive density anomaly body. (**c**) Model of both bodies. Right insets show the perfect data fit between synthetic (pink) and modelled (green) values. The fit is reflected in the fact that it is quite difficult to distinguish between the pink and green symbols because they are superimposed. Growth-dg and Matlab (https://www.mathworks.com) softwares were used to create this figure.

Case i is the most favourable to the inversion routine: large number of gravity observation locations, exact (no error) data, anomalous mass corresponding to density contrast of only one sign (negative in this case), and anomalous body with rounded shape. The inversion method includes a regularization condition to combat the inherent non-uniqueness of the problem (see Eq. (9) in the “Methods” section). This regularization tends to produce rounded, compacted source bodies, which works well with our synthetic example of a rounded body. It is possible to use very low *λ* and *R* values (cf. the Methods section) without introducing artifacts into the recovered model. For values *λ* = 6 and *R* = 1.3% we get a nearly perfect data fit and a model (Fig. 3a) very similar to the synthetic model. The adjusted offset is 500 μGal, average depth is −2026 m and the obtained average density contrast is −15 kg/m^3^.

Case ii is less favourable than case i. The anomalous body corresponds to only a single sign of density contrast, and has an exact data in a large number of points (dense grid). But the simulated body is not rounded, it has right-rectangular T-shape. For values *λ* = 12 and *R* = 3.5% we get a model (Fig. 3b) where the upper regions (more sensitive to the observation data) appear very similar to the original model, but the deeper zones (less sensitive) suffer from the effects of regularization. The adjusted offset value is 501 μGal and resulting density contrast is 13 kg/m^3^ which is very similar to the input values.

Case iii includes a new drawback: the combination of relatively closely spaced positive and negative anomalous density source bodies. In this case the similarity with the original synthetic structure is still good, but slightly worse than that corresponding to the isolated single sign density contrast cases. For values *λ* = 13 and *R* = 3.5% we get a well recovered model (Fig. 3c), but with some additional distortion in the areas where the positive and negative masses are close together, and greater distortion in areas less sensitive to (i.e. further away from) the data. The adjusted offset value, 502 µGal, is again a very close match to the original.

**Figure 4:**
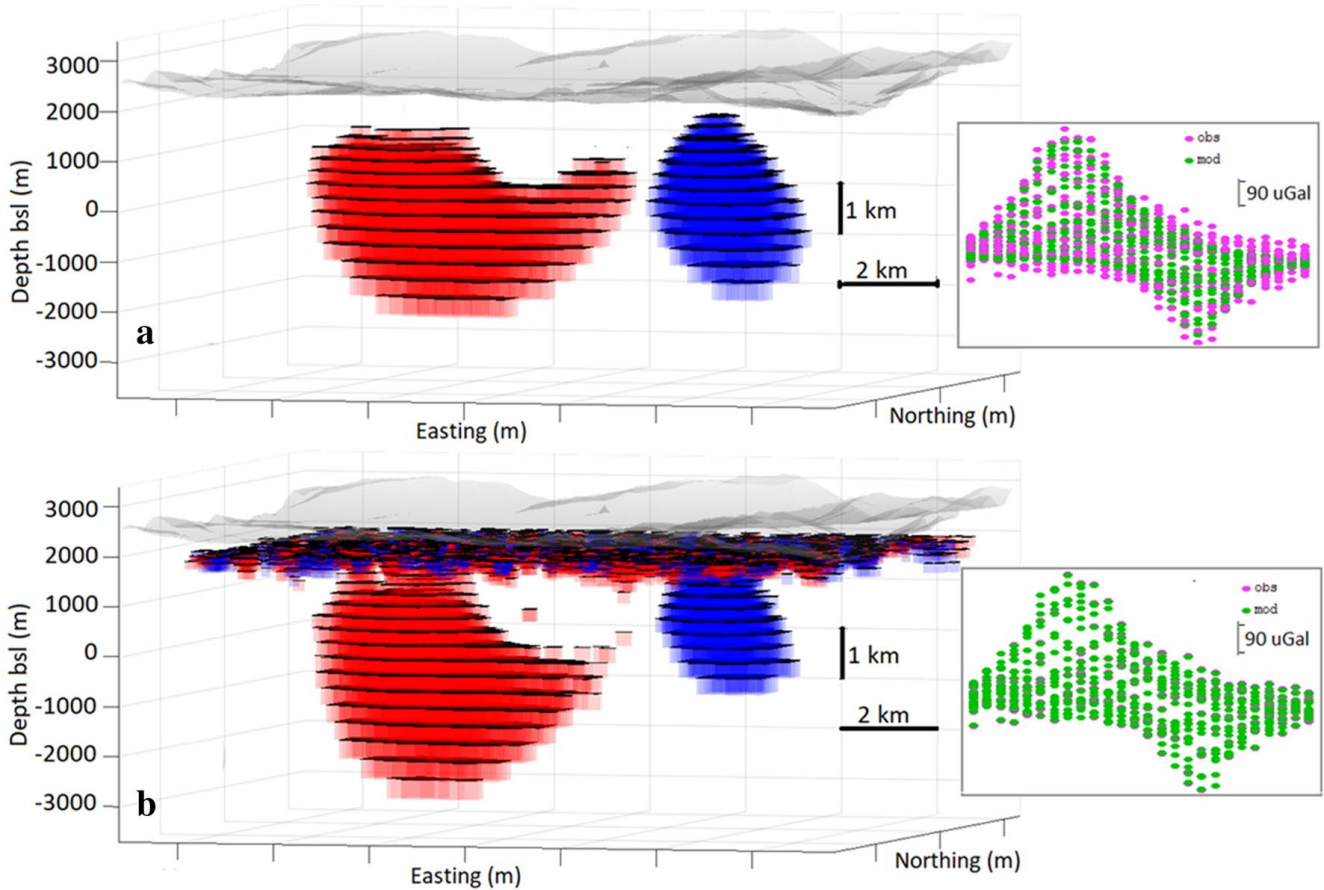
Adjusted models for synthetic inexact data over a large set of data points, Scenario iv. (**a**) High λ value, filtering the noise. (**b**) Low λ value and high R values, creates some shallow artefacts. Growth-dg and Matlab (https://www.mathworks.com) softwares were used to create this figure.

Case iv is closer to that encountered in field surveys. We assume here that the data include a Gaussian noise with standard deviation 37 μGal (about 25% of the standard deviation, 142 μGal, of the synthetic gravity data). Obviously, now the modelled values do not fit 100% of the synthetic data. In this case the method fits the autocorrelated signal in the data and filters the noise as final residuals. This observational noise also causes a certain deterioration of the resulting model, especially in the less sensitive areas (deep and peripheral areas). We take again *R* = 3.5%, but now we consider a much larger smoothing value, *λ* = 90, to help with filtering the noise and avoiding short wavelength artifacts corresponding to the noise. This produces stronger smoothing of the resulting model (Fig. 4a) and filters the noise—standard deviation of the final residuals is 35 μGal. Model distortions have been avoided, and the noise has been filtered, but the resulting model is more rounded and simplified. The adjusted offset value in this noisy case becomes 514 μGal, less suitable than in the previous cases but still close to the input value.

Nevertheless, in this case of a larger number of observation points it is possible to keep a low *λ* value (e.g. *λ* = 12), but take a high *R* value (e.g. *R* = 11). In fact, a low λ value allows modelling a nearly 100% of the synthetic data (final residual order of 2 μGal). It helps recovering the model but also attempt to fit noisy data resulting in very shallow artifacts (Fig. 4b), as corresponding to reproduce this uncorrelated, very short wave component of the synthetic data. In a real life case with low number of sparse data a 100% data fitting gives rise to fictitious bodies at any depth, which can interfere with true objects. In this general case it is necessary to smooth.

**Figure 5:**
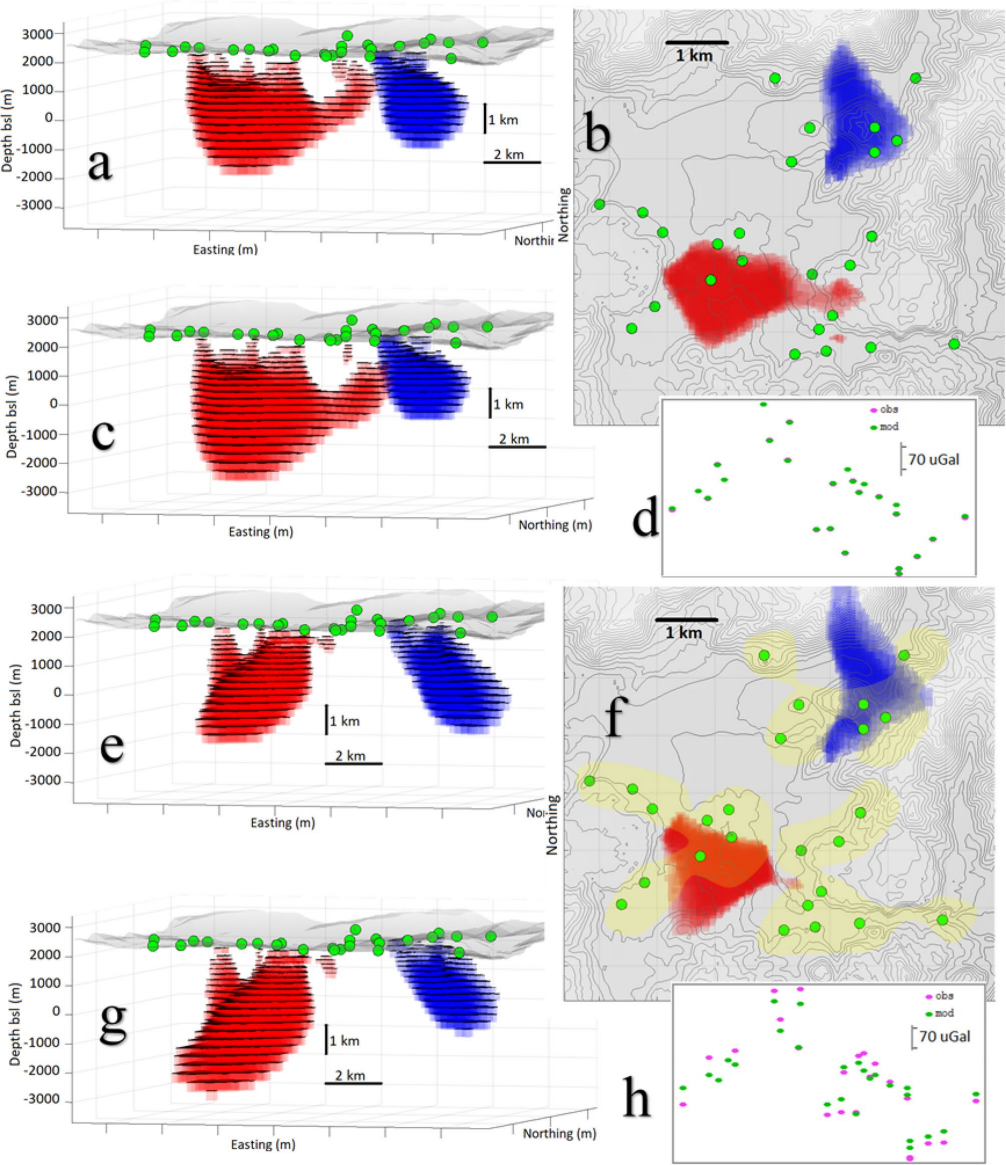
Inversion models for synthetic data over a very small set of data points (Scenarios v and vi). Perspective views, plan views and data fit. (**a–d**) exact data. (**e–h**) inexact data (noise level about 25%). (**a**,**e**) show results allowing offset fitting. (**c**,**g**) show results not allowing offset fitting. Yellow area in panel (**f**) points out proximity to data points and higher model confidence area. Growth-dg and Matlab (https://www.mathworks.com) softwares were used to create this figure.

Case v corresponds to a more realistic conditions encountered in field studies, with very few terrestrial gravimetric stations. Indeed, the repetitive measurement acquisition process is somewhat time consuming and usually not more than two or three dozen quality re-observed stations are available per observation interval. This reduction of data creates a loss of information and consequent distortions in the inversion models. Model in Fig. 5 a, b, and d corresponds to *λ* = 12 and *R* = 6. The value of lambda is selected as a higher value, giving rise to a nearly 100% fit (standard deviation of final residuals is 3 µGal). Adjusted offset becomes 517 μGal, less fitting than in the previous cases, but still within a few percent of the actual value. This model recovered from 24 data points, reflects well the existence and location of both the negative and positive bodies but the geometric details are unreliable as there are not sufficiently close data to provide constraints. Figure 5c corresponds to a similar modelling, but without fitting of the offset value (which is assumed just as 500 μGal). In this case the recovered model is closer to the original than when the offset value is estimated.

Finally, case vi, corresponding to sparse data including now (Gaussian) uncertainty whose standard deviation is about 25% of that corresponding to the synthetic gravity values. Models represented in Fig. 5e–h were obtained with *λ* = 45, *R* = 5. Figure 5g corresponds to include an offset fitting and Fig. 5g shows the results obtained without offset fitting.

Cases v and vi require necessarily the application of high lambda values. In fact, the scarcity of information corresponding to the use of low number of data points, and even more so if it is data with additional noise, increases the uncertainty and this gives rise to distorted recovered models (with respect to the original structure). Low lambda values (nearly 100% data fitting) improve data fit but create model distortions by fitting noise. High lambda values cause higher residuals, but the model (enough simplified in shape and density change value) shows the part of the anomalous structure most supported by the data, especially for those areas closest to the data location.

Distortions are not due to the inversion approach. They are due to the scarcity of information provided by the few available data (in a problem that already involves inherent ambiguity) and cannot be fully eliminated. However, it is possible to reduce potential distortions. First, the use of high *λ* values and low *R* values allows to obtain very essential structures, clearly supported by the data, but with simple geometries (and small density contrast values). On the other hand, in more developed models it is possible to identify some suspicious structures. In fact, these possible distortions occur preferably in areas of low sensitivity (far from the data points).

In Fig. 5f we have denoted the highest sensitivity and therefore most reliable areas, i.e. closest to the data points with yellow (see “Methods” for description of how sensitivity is calculated). Outside these yellow areas, the geometries of source structures are suspicious. Therefore, it is clearly necessary to assess the results within a framework of sensitivity. The graphic outputs of the Growth-dg code (see e.g., Figs. 7 and 8) include information about sensitivity. It is carried out by using parameter *q*_*j*_, given by Eq. (6) in “Methods” section, as a sensitivity value.

For cases v and vi, even considering the results are more approximations to than exact representation of the original anomalous bodies, they give us a very useful information on the buried sources which normally would be unknown density structures.

### Test case: Laguna del Maule volcanic field

**Figure 6:**
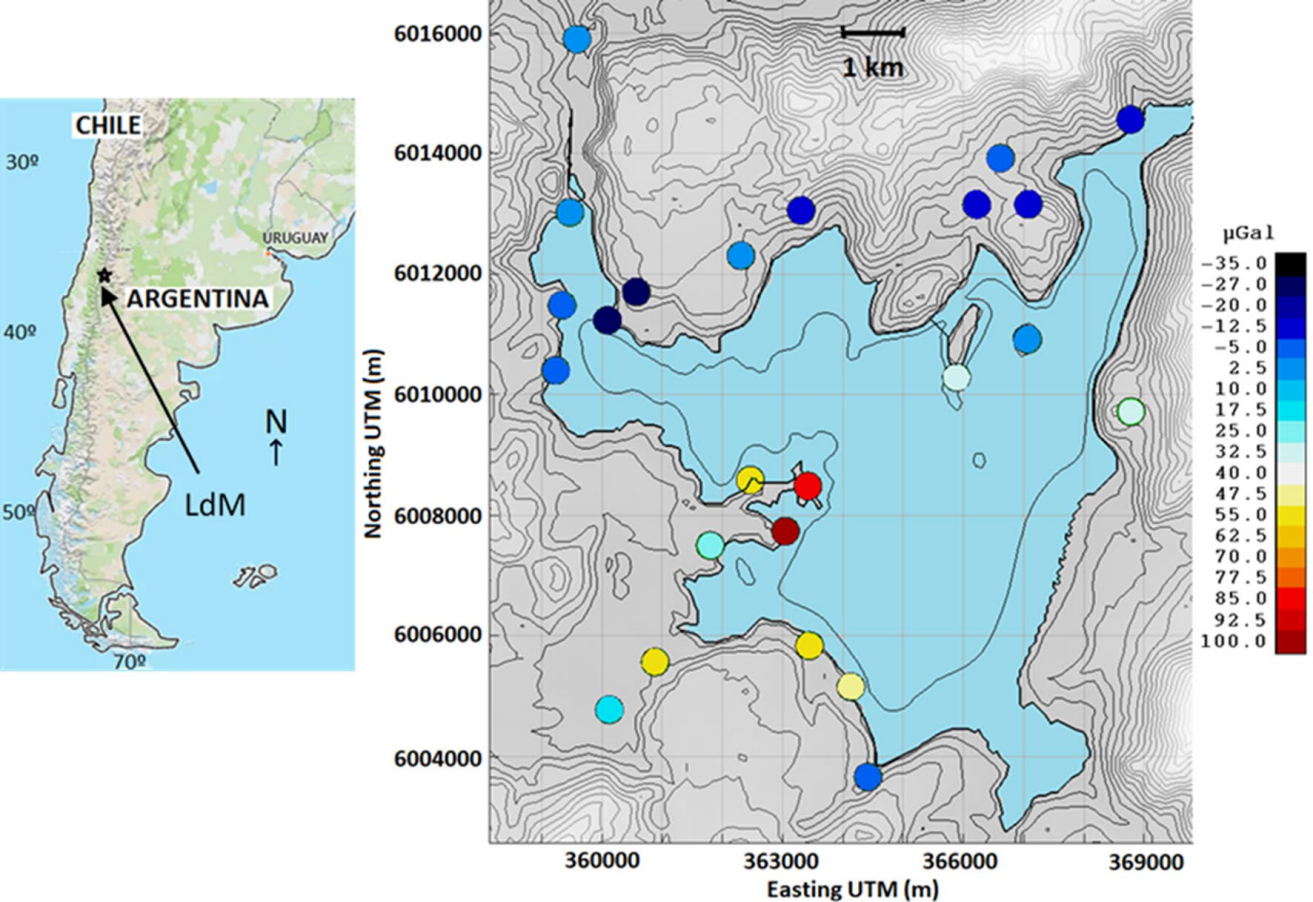
Location of LdMvf. Map of location of LdMvf in the Andes Mountains (left), and more detailed topography of the area with the location of the 24 gravity observation points around the lake (about 2200 m altitude) and residual gravity changes 2013–2014. Matlab (https://www.mathworks.com) software was used to create this figure.

The Laguna del Maule volcanic field (LdMvf) is a large silicic multi-vent volcanic field located in the Andean Southern Volcanic zone (Fig. 6). It is underlain by a shallow large magma reservoir^35–37^. Since 2007, widespread deformation^38,39^ at rates greater than 20 cm/year^40^ have been observed and modelled by an inflating sill^41,42^ at about 3 km below sea level (5 km below surface). The field is characterized by a 19 mGal Bouguer gravity low, centred over the deformation source, caused by a shallow, crystal-poor, volatile-rich, silicic magma system overlying the sill^43,44^.

Precise gravity observations were taken at a network of 35 gravity benchmarks over the area at 4 epochs^45^: April 2013, January 2014, March 2015 and February 2016. The daily observed gravity measurements were corrected for linear gravimeter drift and Earth and ocean tides using GTools^46^. The gravity measurements for each epoch were corrected for the effect of lake level changes between surveys (hydrological correction).

We consider as a test case the precise gravity observations for period 2013–2014 at a network of 24 gravity benchmarks around the lake (Fig. 6). The gravity changes were corrected also for the effect of elevation changes at gravity benchmarks^45^ using the DITE correction, see “Methods” section. For this period the maximum uplift reached 21 cm and maximum residual gravity change reached 97 μGal. The residual gravity changes at benchmarks for the period 2013–2014 are shown in Fig. 6.

**Figure 7:**
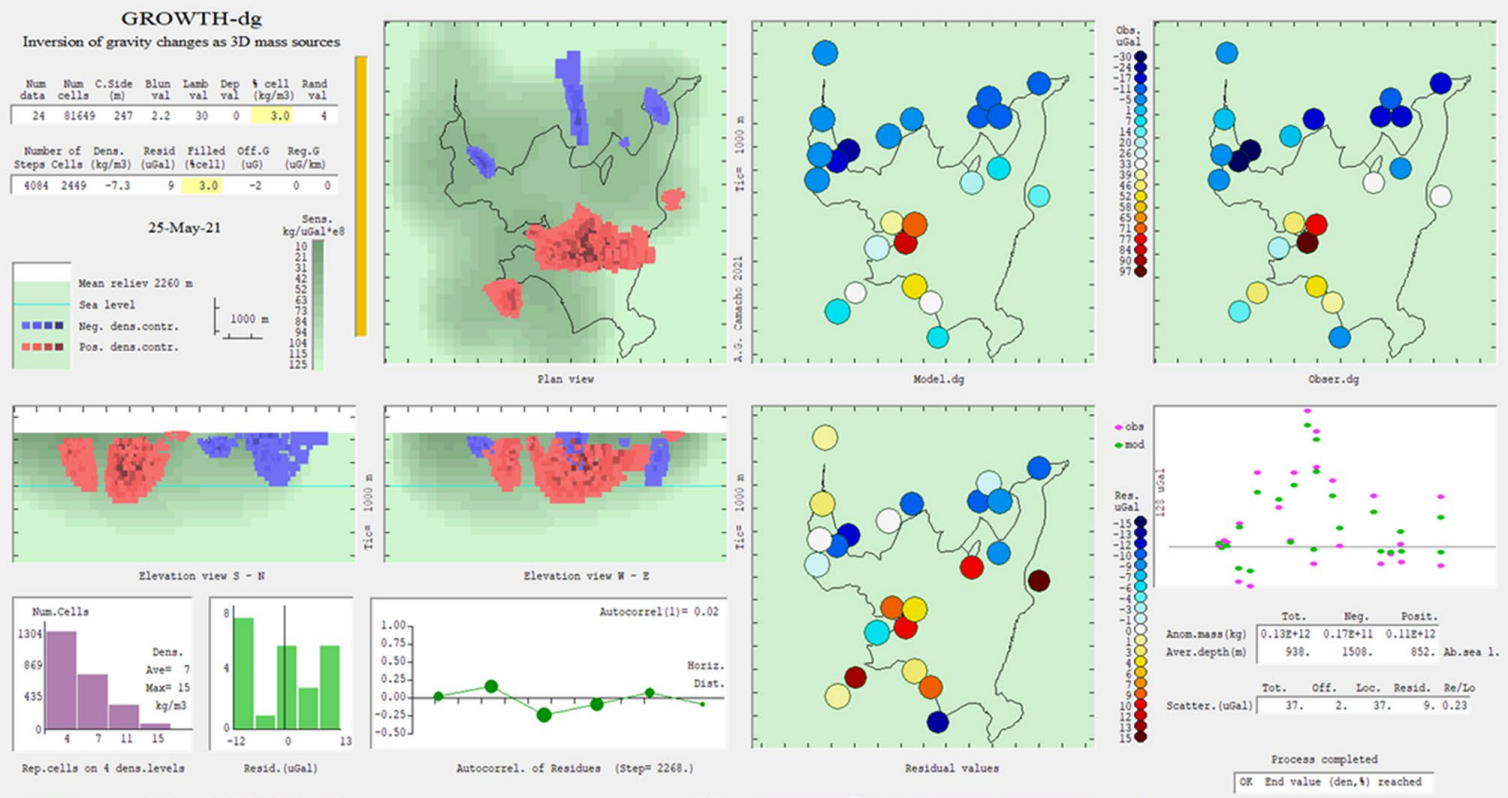
Running screen throughout the inversion process. For each step, it shows: model growth, data fit, model values, residual values, density steps, histogram of residuals and autocorrelation of residuals. Growth-dg software was used to create this figure.

**Figure 8:**
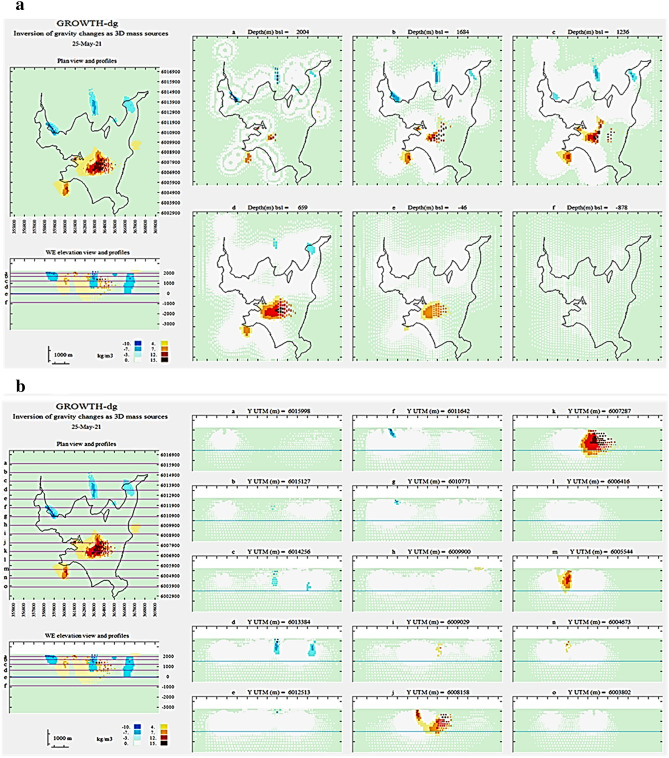
Some running screen examples. Several horizontal (**a**) and vertical (**b**) sections of the resulting model for density changes as shown at the end of the execution of Growth-dg (depths below sea level). Growth-dg software was used to create this figure.

The input gravity data are organized in a text file (“grad.txt”) that includes UTM coordinates (m), elevation (m) of gravity stations and residual gravity changes (μGal) at these stations. This file is the data input for the Growth-dg inversion routine. Figure 7 shows the running screen during the execution of the Growth inversion, while Fig. 8 displays the resulting inversion model with horizontal slices and vertical sections. Both show the results obtained for the 2013–2014 gravity change data.

**Figure 9:**
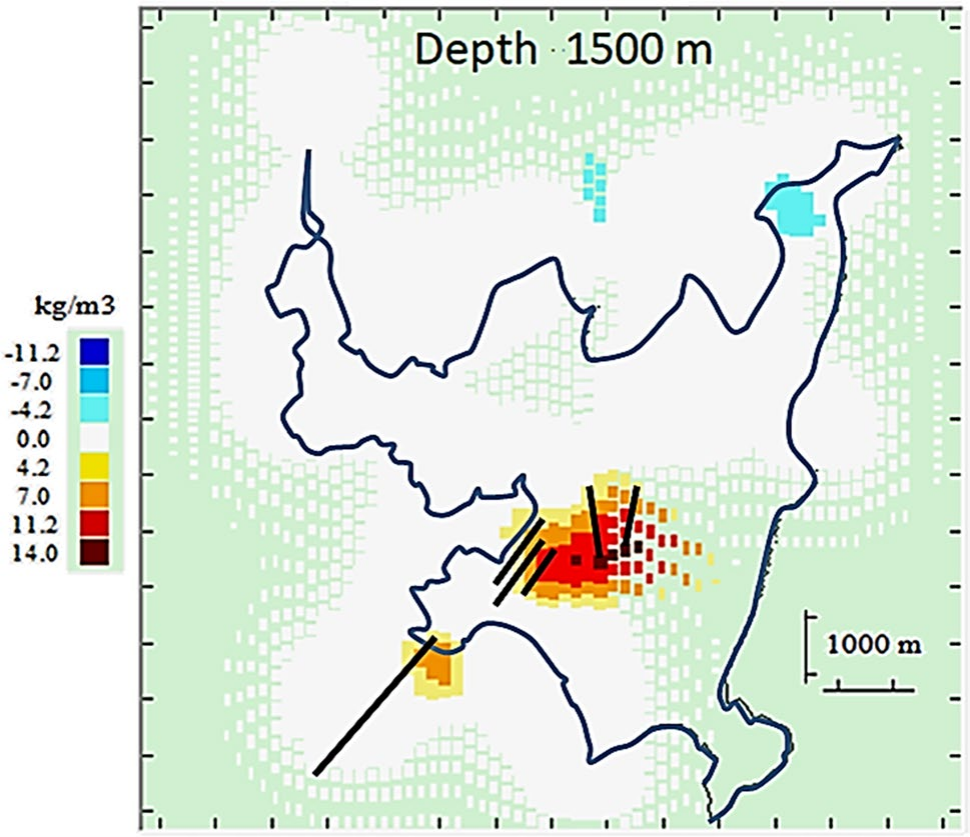
Horizontal section of the model for density changes at a depth of 1500 m below surface. Continuous white area corresponds to high sensitivity (and model reliability) Green and discontinuous white areas means low sensitivity (and low model reliability). Black lines show location of known local fault traces^47^. Growth-dg software was used to create this figure.

The subsurface volume partition is composed of 70,661 cells with average horizontal dimension of 249 m (see Fig. 7). Then we accept the default value *R* = 3, and we take, after some trials, a value *λ* = 30. Results are summarized in Fig. 9 showing the anomalous density body at a slice at a depth of 1500 m below sea level.

## Discussion

The proposed inversion approach models imprecise residual gravity data observed in a small network. It works in a 3D context and without requiring a particular starting model or hypothesis. Some characteristic of this method are: (1) non-linear fit of the 3D geometry described as aggregation of small filled cells, (2) regularization conditions by model smoothing given by a balance parameter *λ*, (3) iterative refilling of cells according a prescribed number of density levels given raise to non-homogeneous models, (4) optional determination of a gravity offset value, and (5) control for outlier values.

The method is developed in a software that can be easily applied and provides many figures for understanding the inversion process and output. The software offers suitable default values for all the parameters. However only two parameters are critical for modelling: a model volume size (as % of the available volume) and the smoothing parameter *λ*. For this last parameter a trial approach coupled with prior user knowledge of the survey target, is the best way to determine a suitable value. High *λ* values provide simple models, small in size and with rounded shapes but data fit is poor. Low *λ* values provide small resulting residuals but resulting inverse model become too large and geometrically complex.

The sparsity of data points in 4D gravity surveys creates a shortage of information about the field of gravity changes and any overly ambitious inversion approach could produce distortions in the resulting models. The autocorrelated component (signal) contained in the data is poorly defined, and much of the information contained in the data (even though it is of proven quality) remains insufficiently correlated and becomes local noise. This sparsity of information gives rise to models with distortions if incorrect inversion parameters are used, such as fictitious artifacts for low *λ* values, or overly simplified models for high *λ* values. In order to interpret models with low spatial autocorrelation between the data values (with respect to horizontal distance), it would be necessary to have some additional information on the characteristics of the subsurface. In the absence of this information, we must be cautious and opt for high *λ* values and low *R* values, which fit the autocorrelated component of the data and offers concentrated models. It produces models with less fit to the gravity data, but the obtained simple model (rounded shape, small density contrast) is more reliable as core of the anomalous structure. Given the often-poor signal to noise ratio of gravity change values this is an appropriate approach and prevents over-fitting noisy data.

This methodology and software can be applied to present and past studies of gravity changes in volcanic areas to get independent models with a mostly non-subjective base. It is also applicable to datasets from hydrogeology, geothermal, geotechnical studies that are characterised by low numbers of observations.

Free body inversions, such as we present here, compliment analytic solutions, which often require laborious trial and error to determine the optimal geometry (vertical vs horizontal source, etc.). Analytic solutions have the advantage in that they are well determined, but it must be assumed an a priori geometry for the source and often those geometries are unlikely to be encountered in nature. By comparison free-body inversions provide the ability for approximating the true shape of the mass or density change region to be recovered.

Both the original Laguna del Maule inversion result^45^ obtained using analytic solutions and our new free body approach show a vertically elongate body subparallel to the Troncoso Fault. The optimal analytic solution of a vertical prism was obtained after exhaustive testing of many source geometries, whereas the free body solution was derived automatically in a single inversion run. Additionally, the free body inversion shows an east–west elongation at the north end of the body, that a single fixed geometry inversion is unable to account for.

The new geological interpretation resulting from the free body inversion suggests that additional faults, including those mapped by Peterson et al.^47^ are involved in the accumulation of hydrothermal fluids, not just the Troncoso Fault. The low density change recovered by the free body inversion suggests aqueous fluid accumulation in permeable rocks along fault traces. Our new model implies that the region stressed by active magma intrusion at depth is broader than originally thought and has implications for present day and future faulting and doming beneath the lake that is occurring as part of on-going magma injection.

## Methods

### Residual gravity changes as input data for the inversion procedure

The input gravity data is the gravitational effect of the sought sources of temporal mass and density changes expressed in units of µGal. By mass changes we mean addition or removal of mobile masses to/from the subsurface volumetric domain of the model. By temporal density changes we mean the changes in density of the masses originally present within the subsurface model domain.

The observed gravity changes must be first corrected for all signal components other than those associated with the processes of interest, such as atmospheric and tidal effects, instrumental drift, hydrological effects^12,14,16,48^ and surface mass changes^49^. If surface deformation (uplift or subsidence) accompanies the observed gravity changes, they must be properly corrected for the gravitational effect of the surface deformation. This correction is given in terms of the deformation-induced topographic effect^50,51^ and is to be evaluated by Newtonian volumetric integration. The gravity changes are affected also by the gravitational effect of inner (subsurface) deformations. Those, however, are not directly observable, unlike the surface vertical displacements, and their effect cannot be therefore computed and applied as a correction to observed gravity changes. It can be only estimated or modelled^50,52^. The application of all the above corrections to observed gravity changes results in the residual gravity changes that are used as input data in our inversion process.

Tidal, barometric and observational effects (drift, calibration) can be externally accounted for. However, hydrological effects can amount tens of microgals and are often very localised^1^ (60% of the whole hydrological effect is up to 1 km). They are almost impossible to mathematically simulate, and can give rise to high uncorrelated noise (which is not an error) that would require additional information for possible interpretation.

## Methodology of gravity changes inversion

### Principles

As in many other gravimetric inversion methods^25,32,53^, our methodology is based on the adjustment of free-geometry structures described by aggregation of cells filled with anomalous density values. The sensitive volume (according to a minimum value of average vertical attraction upon the observation stations) of the subsoil is divided into a partition composed by *m* (tens of thousands) prismatic cells. The geometry of the vertical rectangular parallelepiped is chosen because it facilitates the routine of setting the partition and has a relatively easy expression for its vertical attraction at an external point. In fact, the gravity attraction, *A*_*ij*_, at the observation point *P*_*i*_*(x*_*i*_*,y*_*i*_*,z*_*i*_*)*, due to the *j*-th prism, for unit density, is given by Pick et al.^54^:

1 $${A}_{ij}=-G\left[\left[\left[x\mathrm{ln}\left(y+\sqrt{{x}^{2}+{y}^{2}+{z}^{2}}\right)+y\mathrm{ln}(x+\sqrt{{x}^{2}+{y}^{2}+{z}^{2} })+z arctg\frac{z \sqrt{{x}^{2}+{y}^{2}+{z}^{2}}}{x y}\right]\begin{array}{c}{u}_{2}^{j}-{x}_{i}\\ {u}_{1}^{j}-{x}_{i}\end{array}\right]\begin{array}{c}{v}_{2}^{j}-{y}_{i}\\ {v}_{1}^{j}-{y}_{i}\end{array}\right]\begin{array}{c}{w}_{2}^{j}-{z}_{i}\\ {w}_{1}^{j}-{z}_{i},\end{array}$$

where *G* is the gravitational constant, the edges of the *j*th prism are parallel to the reference axes, and the limiting coordinates for its volume are: ${u}_{1}^{j},$ ${u}_{2}^{j}$ for the x coordinates, ${v}_{1}^{j},$ ${v}_{2}^{j}$ for the y coordinates and : ${w}_{1}^{j}$ ,${w}_{2}^{j}$ for z. Matrix ***A***, with components *A*_*ij*_, is the design matrix of the physical configuration of the problem, and the observation equations of the problem would initially be:

2 $$\Delta {g}_{i}-\sum_{j\in J}{A}_{ij} \Delta {\rho }_{j}={v}_{i}, i\hspace{0.17em}=\hspace{0.17em}1 \dots n,$$

where, $\Delta {\rho }_{j}$ is the (negative or positive) anomalous density value for *j*-th cell, *J* is the set of indexes corresponding to those cells filled with no-null density values, and *v*_*i*_ are residual values. In order for the set of unknowns to be balanced, we adjust the sizes of the cells so that for all of them their average (square) gravimetric effect on the network of *n* stations for unit density, *E*_*j*_:

3 $${E}_{j}=\sqrt{\frac{\sum_{i=1}^{n}{A}_{ij}^{2}}{n}},$$

is approximately the same for *j* = *1,…,m*. Those cells located shallow and close to the observation stations will be small size and the deep or peripheral cells will have large sizes.

In these circumstances, there are two main approaches to the problem: (a) try adjusting the density contrast values $\Delta {\rho }_{j}$ all across the subsurface volume, it would be the linear approach, and (b) try determining a free geometry (a set *J*) for a prescribed density values , negative and positive $\Delta {\rho }_{j}^{-}$ and $\Delta {\rho }_{j}^{+}$, it would be the non-linear approach. Approach (a) has the advantage of linearity, but it produces blurry or diluted models, which do not allow for clear identification of shapes and other geometrical aspects. Advanced methods allow to get definite geometrical properties by adding to Eq. (2) some constrains about compactness^25,55,56^. Approach (b) has the advantage of provide clear geometrical properties, but the non-linearity requires a more complicated resolution process. Furthermore, assuming predetermined density values involves some restriction. The purpose of this methodology is to propose a non-linear inversion, type (b), but allowing the densities to have different values, adjusted in the inversion process, rather similar to type (a). We present the methodology following several steps.

### Observation and regularization equations

The least square minimization of residuals *v*_*i*_, matrix written as

4 $${{\varvec{v}}}^{T}{{\varvec{Q}}}_{{\varvec{D}}}^{-1}{\varvec{v}}=min,$$

is a usual model fitness condition for solving problems with Gaussian data uncertainties corresponding to a priori covariance matrix ***Q***_***D***_. *T* means transposed matrix. But, as it is well known, it is not enough to provide unique solution for the inverse gravity problem^57^ (as usual for potential problems). For instance, for any solution we can obtain an equally fitting solution, but more complex one, by adding a “checker board” of positive and negative close density elements within the subsurface volume. On the other hand, the presence of high levels of observational signal/noise (even up 30% for gravity changes) requires to consider some control allowing to invert the signal and discard the noise (denoting by noise the not autocorrelated part). Both problems are solved by adding some regularization or smoothness conditions. Indeed, once the density contrast has been set, imposing that the solution verifies a limitation to its size or complexity allows to obtain solution uniqueness. The minimization condition (Eq. 4) is now substituted by a mixed minimization condition, composed by the model fitness and a model “smoothness” given by the *l*_*2*_-minimization of total anomalous mass:

5 $${{\varvec{v}}}^{T}{{\varvec{Q}}}_{D}^{-1}{\varvec{v}} + \lambda {{\varvec{m}}}^{{\varvec{T}}}{{\varvec{Q}}}_{M}^{-1}{\varvec{m}}=min,$$

where column vector ***m*** correspond to the model anomalous density values ${\Delta \rho }_{j}$ (positive and negative) for all *m* cells in the subsurface partition, ${\varvec{m}}={({\Delta \rho }_{1},\dots ,{\Delta \rho }_{m})}^{T}$, ***Q***_***M***_ denotes the a priori covariance matrix for uncertainties of the parameters of the model. *λ* is a factor for the selected balance between the fitness and the smoothness of the model. Matrix ***Q***_***M***_ plays the role of giving cells at different depths equal probability to enter into the solution with a nonzero value^58^. We set a diagonal matrix ***Q***_***M***_ with elements *q*_*j*_ (*j* = 1,…,**m**) corresponding to *j*-th cells, centred in *X*_*j*_*, Y*_*j*_*, Z*_*j*_ and with volume *V*_*j*_, as given by:

6 $${q}_{j}=\frac{{V}_{j}}{n}\sum_{i=1}^{n}\frac{\left|{z}_{i}-{Z}_{j}\right|}{{r}_{ij}^{3}},$$

where *r*_*ij*_ is the distance between cell *j*th *(X*_*j*_*,Y*_*j*_*,Z*_*j*_*)* and benchmark *i-th (x*_*i*_*,y*_*i*_*,z*_*i*_*)*. However, the present methodology, independently of this covariance model matrix, produces fitted structures at adequate depths (see “Fitting within a scale factor” below).

Parameter *λ* plays a key balance role. It allows to regulate the proportion of the effects of the minimization of residuals and the minimization of the model magnitude. High *λ* values provide simple models, small in size and with rounded shapes. It is a conservative criterion however data fit could be poor. Conversely, low *λ* values provide small resulting residuals where some of the observational noise can be fitted. However, resulting inverse model become too large and unjustifiably geometrically complex. The synthetic example presented illustrates the effects of considering a high or low value. A suitable criterion for choosing the *λ* value comes from the autocorrelation analysis of the resulting gravity residual values. An optimal modelling would be one that adjusts almost 100% of the correlated signal and none of the observational noise. It can be verified by the subsequent autocorrelation analysis. This works well for CBA data (many observation data with high signal to noise ratio). However, in the case of gravity changes, the scarcity of data and the high proportion of noise make it difficult to fully application. We recommend a rather conservative application of the criterion of the autocorrelation analysis (for example, do not exceed the 80% adjustment of the correlated signal). Finally, always rely on the visualization of the geometry of the resulting model to confirm geological plausibility of the model.

### Offset gravity value

Survey gravity values are mostly observed with relative instruments, more suitable for precise field work than larger absolute gravity meters. Gravity changes are usually estimated by assuming no changes for some network point far from the active area. For well-delimited geodynamic processes this hypothesis is usually fully correct. However, in a lesser known site there may be doubts about the stability of the outer reference point. An unknown variation in the reference point would result in a uniform variation, or offset *∆g*_*off*_, in the points of the network. Then Eq. (2) becomes:

7 $$\Delta {g}_{i}-\Delta {g}_{off}-\sum_{j\in J}{A}_{ij} \Delta {\rho }_{j}={v}_{i},i\hspace{0.17em}=\hspace{0.17em}1 \dots n,$$

The existence of an undetected offset value in the gravity data could produce a fictitious very deep anomalous structure in the inversion results. This suggested fit prevent it.

Within the non-linear context Eqs. (7) and (5) would be the modelling equations. Anomalous density values would be prescribed a priori (see below), and unknown parameters would be (1) *∆g*_*off*_ and (2) the set *J* that identifies the cells filled with the non-null density values. Set *J* represent the anomalous geometry as aggregation of filled cells.

### Fitting within a scale factor

Equation (7) correspond to a clear non-linear context (at least for *J*). This is an additional complication. Without approximate initial solutions, maybe the best inversion approach is the exploration of the model space^59^. However, in this case the model space M is made up of all possible combinations of *m* cells, $\sum_{l=1}^{m}C(m,l)$. For *m* usually equal to tens of thousands the approach would be excessive.

Fortunately, in the gravity (and other potential fields) inversion problem there is an interesting alternative. The geometry of the anomalous model can be elaborated in a growth process. For each *k*-th step of this process, only a new cell is filled with the prescribed density and aggregated to the previous structure. So, the full exploration is substituted by successive explorations to select only one new cell from *m* (or *m–k)* possible cells. It is a much more affordable approach.

Mathematically, it is carried out by means of the introduction of a new parameter: a scale factor *f*. We assume only one a priori value *∆ρ* for density contrast everywhere, which can appear with negative or positive signs. For any intermediate step, the model is not fully developed (density contrast *∆ρ*) but it remains almost homothetic with the final model, and must verify the equations to scale. The model equations are now written, for each *k*-th step of the process, for an intermediate density value ± *f* as:

8 $$\Delta {g}_{i}-\Delta {g}_{off}-f(\sum_{j\in {J}^{+}}{A}_{ij} +\sum_{j\in {J}^{-}}{A}_{ij} )={v}_{i}, i\hspace{0.17em}=\hspace{0.17em}1 \dots n,$$

9 $${{\varvec{v}}}^{T}{{\varvec{Q}}}_{D}^{-1}{\varvec{v}}+\lambda {f}^{2}{{\varvec{m}}}^{{\varvec{T}}}{{\varvec{Q}}}_{M}^{-1}{\varvec{m}}=e=min,$$

where positive and negative density values $\Delta {\rho }_{j}$ in Eqs. (8) and (9) have been expressed as ± *f* and where *J*^+^ and *J*^*-*^ are the set of indexes corresponding to those cells filled with positive and negative density values.

So, for this *k*th step, *k* − 1 cells have been previously filled, and they fit Eqs. (8) and (9) by means of a value *f*_*k*−1_ of the density scale factor. Now the approach explores all candidate cells looking for selecting and filling only a new one. For each candidate cell the process carries out a linear least squares fit of values for *∆g*_*off*_ and *f* in Eq. (8). With these adjusted values, we can evaluate the value *e* of expression (9). That cell minimizing *e* in Eq. (9) is selected to be filled and aggregated. The growth process continues with *f* and *e* values smaller and smaller according to the model grows by aggregation of cells. The process ends when the scale factor reaches *f* = *∆ρ*, or conversely when it is not possible to find a new cell with values *f* and *e* smaller than those of the preceding step. In this last case the last *f* value would be the resulting model density.

### Heterogeneous models and process end

The former approach is based on assuming a single density contrast ± *∆ρ* everywhere. This is a simplifying hypothesis but may create unrealistic results. Furthermore, the gravimetric inversion without additional geological information allows to find out geometric properties (location, shape) on the anomalous structures, but it hardly allows to estimate realistic values of the density contrasts, be it one or several. However, it is useful to be able to handle or adjust various density contrasts, especially when the anomalous bodies to be modelled may include areas clearly differentiated by their different anomalous density. This could reveal different components in the bodies detected from the spatio-temporal changes of gravity.

Within the previous methodological approach, the option of several adjustable density contrasts can easily be included through the possibility of reconsideration of the cells. In the case of anomalous homogeneity, only the empty cells are considered in each step as possible candidates to be filled in and added. However, if in each step we consider all cells (filled or not) as possible candidates, it will happen that some cells will be re-filled and their anomalous density will become two (or three or four …) times the basic density. This idea of revisiting the cells has the additional advantage of improving the quality of the modelling, since the new cells are less dependent on the initially filled ones.

**Figure 10:**
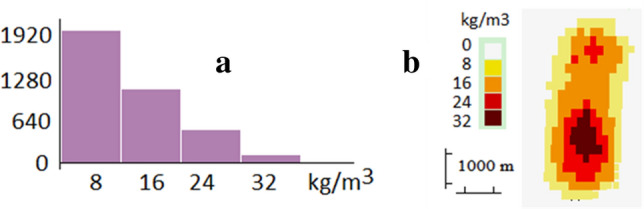
Example of distribution of filled cells keeping Eq. (10). (**a**) Number of cells that have been filled a number of times (from 1 to 4) vs. density of cells (8 kg/m^3^ for filling once, 16 kg/m^3^ for filling twice, etc.). (**b**) Spatial aspect of the model verifying Eq. (10) with a suitable distribution of regions with 1 to 4 times filling. Growth-dg software was used to create this figure.

However, this option of free reconsideration for the cells has the disadvantage that it gives rise to very concentrated patterns (high density) on the main lines of the anomalous structures, with little lateral extension (see the section for synthetic case). To get more suitable models we propose to introduce two additional conditions: (1) Limit the maximum number of cell refills to a value *NR* (for instance, *NR* = 4). It produces models more extended, but with enough information about different density zones. (2) Allow additional refill of any cell only when the proportion of refilled cells keep a prescribed law as, for instance:

10 $$nc\left(j\right)\le nc(1) {\left(\frac{NR-j}{NR}\right)^{2},\mathrm{j}=2 \dots .\mathrm{ NR}}$$

where *nc*(*j*)*,* 1 ≤ *j* ≤ *NR,* is the number of cells *j* times filled. This law has been selected, after some trials, due to its suitable balance of mass in the resulting models. Figure 10 shows an example, with the distribution of cells corresponding to Eq. (10).

Using these density levels, we propose to determine the end of the process when the average density value $\Delta {\rho }_{m}$ for the model densities,

11 $$\Delta {\rho }_{m}=\frac{\sum_{j\in J}{f nr}_{j}}{\sum_{j\in J}{nr}_{j}},$$

reaches the prescribed value *∆ρ.*

In the common case of not having a priori values *∆ρ* for mean anomalous density, we propose to use an alternative end condition and then a determination of a mean density contrast. This condition refers to the increasing relative size of the resulting model, expressed by the proportion between the total number of filled cells and the total number *m* of available cells:

12 $$\frac{\sum_{j=1}^{NR}nc(j) }{m}\approx R.$$

After some trials we suggest values *R* close to 1%–5% as suitable end condition (in absence of other information). However, these data-poor inversion models of density variations hardly admit generalization or regulation. Perhaps the most appropriate thing is to carry out various tests and observe the evolution of the models. For this reason, the calculation program that is presented here shows various graphs and figures on the screen that can help to choose both the value of the smoothing $\lambda$ parameter and the value of the termination *R* parameter.

### Iterative reweighting for robust approach

As previously pointed out, data for gravity changes, despite being values collected with extreme care, could include a high level of noise due to local (mostly hydrological) effects. These local effects would correspond to not correlated components in the data, and theoretically they cannot be properly interpreted based on the data alone. There could even be data that, although of good observational quality, contain particularly high local effects, so that in the total dataset they are outliers. Therefore, we must contemplate this possibility and foresee some process to detect such data and adequately minimize its effect on the inversion. For that we use a robust procedure consisting of iteratively reweighted least-squares solutions by successively assigning small weights to large residuals.

**Figure 11:**
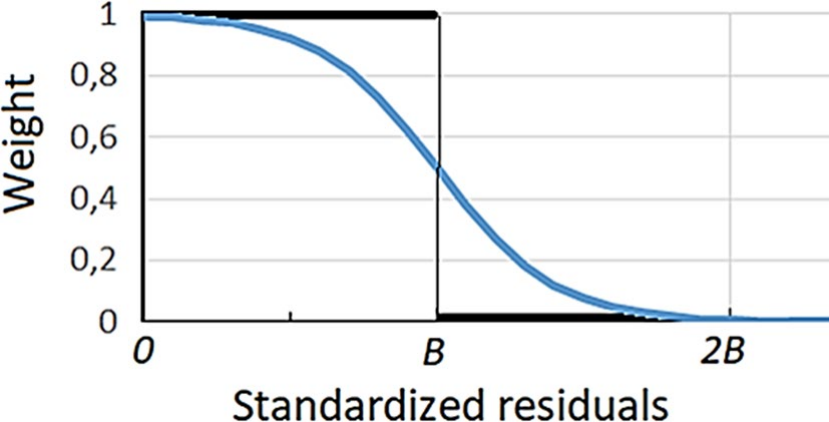
Weighting functions. Step (black) and smoothed (blue) weighting functions used to reduce the effect of data corresponding to high values of the standardized residuals $\frac{{\mathrm{v}}_{\mathrm{i}}}{\widehat{\upsigma }}$ (B blunder value ≈2.1). This figure was created using Microsoft Excel 2016.

Gravity residuals corresponding to random errors would fit a normal distribution. However, some residuals could be not normally distributed. We assume that these residuals would be rare. Then, the part of the combined distribution within ± *B σ*, with *B* blunder value (for instance *B* = 2.5), should be dominated by random errors, and the region >  ± *B σ* where the normal distribution is small should be dominated by a flat distribution of outliers (all sizes of outliers have equal probability). Then outliers could be detected by analysing the final residuals^60^. First, for each step of the growth process, we calculate a robust estimation $\widehat{\sigma }$ of the standard deviation of residuals *v*_*i*_ by means of the mean of the absolute values as:

13 $$\widehat{\sigma }=\frac{1}{0.6745}\frac{\sum_{i=1}^{n}\left|{v}_{i}\right|}{n},$$

where the constant 0.6745 makes $\widehat{\sigma }$ a consistent estimator of the standard deviation in the case of observations contaminated by Gaussian noise. The standardized residual $\frac{{v}_{i}}{\widehat{ \sigma }}$ computed with this deviation estimate are used to determine a weight ${w}_{i}$ for each data value *dg*_*i*_ , *i* = 1,…,*n*, to be used in the next step of the growth process. A possibility would be to set the ${w}_{i}$=1 in the region inside ± *B* $\widehat{\sigma }$ and ${w}_{i}$=0 in the region outside ± *B* $\widehat{\sigma }$, but this unit step function would eliminate the outliers definitively in the process. A less radical condition is to use a smoother filter. We propose a logistic function similar to that one usually used to smooth the Heaviside step function:

14 $${w}_{i}=\frac{1}{1+{e}^{c {t}_{i}}},$$

where ${t}_{i}=\frac{{v}_{i}}{\widehat{ \sigma }}-B$, and where a larger value of coefficient c corresponds to a sharper transition at *t* = 0. The smoothly tapering function for weighting makes also the convergence of the solution less erratic than with a step function. Taking into account that we are dealing, not with high errors, but with some quality values containing high local noise, we use low values for *c* and *B*, for instance *c* = 4 and *B* = 2.2. Values for the step and the smoothed functions are plotted in Fig. 11 as function of the standardized residuals $\frac{{v}_{i}}{\widehat{ \sigma }}.$

### Additional effects of depth in modelling

In usual geodynamic environment, as for instance the volcanic sites, intrusions of anomalous density would tend to lodge in areas of discontinuity capable of accommodating them. We can assume that such host niches will be sub-horizontal strata. However, according to a general buoyancy criterion, the background areas below the intrusion would be denser than the areas above. In general, terms, the density of the environment would increase downwards. This could affect slightly to the anomalous geometry and can be taken into account in the modelling approach. Indeed, positive anomalous structures will be somewhat displaced or prolonged downwards, while negative anomalies will be somewhat displaced or prolonged upwards. Perhaps the easiest form to apply it in the former approach will be by multiplying the neutral weighting, given by (6), by an additional factor proportional to the cell depth with respect to a mean model depth (*Z*_*M*_),

13 $${q}_{j}(\pm )=(1\pm D \frac{{Z}_{M}-{Z}_{j}}{{Z}_{M}}) \frac{{V}_{j}}{n}\sum_{i=1}^{n}\frac{\left|{z}_{i}-{Z}_{j}\right|}{{r}_{ij}^{3}}$$

where ± corresponds to positive or negative density contrast for the *j*th cell, and *D* ≥ 0 is a factor for greater or lesser effect. For *D* = *0* there is not this down-up effect.

### Code Growth-dg

**Figure 12:**
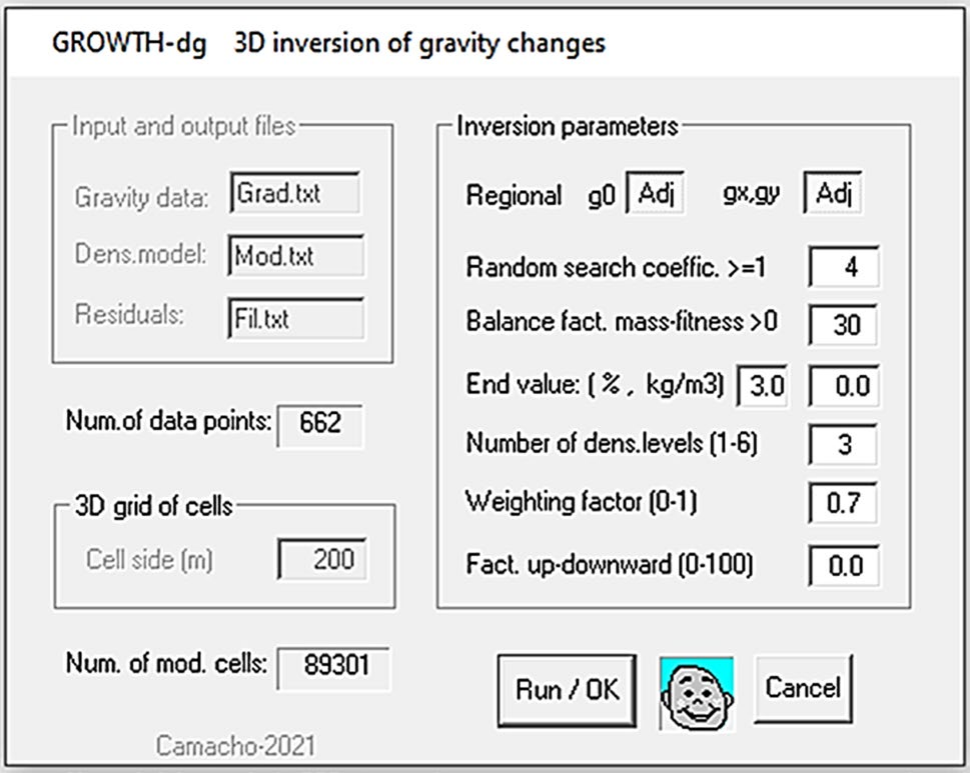
Dialog window. Dialog window for entering the values of the inversion parameters in the program Growth-dg. Growth-dg software was used to create this figure.

The described inversion approach for gravity changes materializes in an immediate application software. It consists of a single Fortran program, Growth-dg, compiled with Intel Visual Fortran, by using a Quickwin Application Project, and is compatibility with the current Microsoft (Windows 10 Pro) operative system. The execution requires an ascii data file with the gravity changes (UTM coordinates, elevation, and gravity changes in µGal) and, optionally, a file “map.bln” with some points for drawing annotation lines. Additional input values (*λ*, *NR*, *∆ρ*…) for running the inversion process are entered using a simple dialog box, which offers default values for all of them (Fig. 12). The entering of values for parameter is done in three steps. In the first step, the program asks to confirm, or to modify, default names of the input and output files. In the second step the program asks to confirm default value for average size (average side length) for the partition cells pre-calculated by the program automatically based on the spacing of the input gravity data, which can be eventually modified by the user. In the third step, once the average cell side is accepted or modified, the program asks to confirm or modify the default values for the inversion parameters (offset, λ, NR, ∆ρ, D,…).

In this software, the exploration of model space *M* (across the available cells for each growth step) referred to in “Fitting within a scale factor” above, can be carried out slowly systemically, or much faster using a random approach. For this last case, the dialog window asks also for the value of the coefficient *r* for random exploration. For each step, *m/r* cells randomly chosen are explored. For *r* = *1* slow systemic exploration is selected.

The main parameters to consider are *λ*, *R* (or *∆ρ)*, balance factor smoothing-fitness and model size (for ending condition), respectively. For *λ* we can accept initially the default value, and then try (observing the model aspect and the distribution of residuals) other values. For *R* we suggest to try values from 1 to 5% (default is 3%). The remaining parameters (offset, *NR*, …) are less important, and the default values are enough good.

Once the input values are prescribed, the running process is automatic. An execution screen (Fig. 5) is displayed permanently showing step by step numerical values and pictures for: model growth (by means of a plan and elevation views of the growing model), data fit, modelled values, residual values, density steps (for heterogeneous models), histogram of residuals and autocorrelation of residuals with respect to their horizontal distance. This set of figures is an interesting tool for inversion of gravity changes, because it can help the user to know how the model is forming, and to better decide the characteristic values of the inversion process.

After the execution is complete, the program then displays several horizontal and vertical sections of the resulting model (Figs. 7 and 8). Finally, two result files are generated. One with the geometric description of the model (cell to cell values) allowing importing into external visualising software and the other with the observed, modelled and residual values for each station.

## Data Availability

Data files and result files used for synthetic test are available and for the real test case from the websites http://gegrage.ucm.es/en/software-en/ and https://github.com/josefern/Growth-dg.

## Code availability

This software (including source code), a user manual and same example files are free available for download from the websites http://gegrage.ucm.es/en/software-en/ and https://github.com/josefern/Growth-dg.
